# Effect modification by region in the associations of *LEP G2548A* and *LEPR Q223R* polymorphisms with statin-induced CK elevation

**DOI:** 10.18632/oncotarget.22506

**Published:** 2017-11-18

**Authors:** Shanqun Jiang, Scott A. Venners, Kang Li, Yi-Hsiang Hsu, Justin Weinstock, Yanfeng Zou, Faming Pan, Xiping Xu

**Affiliations:** ^1^ School of Life Sciences, Anhui University, Hefei, China; ^2^ Institute of Biomedicine, Anhui Medical University, Hefei, China; ^3^ Faculty of Health Sciences, Simon Fraser University, Burnaby, BC, Canada; ^4^ Institute for Aging Research, HSL and Harvard Medical School, Boston, MA, USA; ^5^ Molecular and Integrative Physiological Sciences Program, Harvard School of Public Health, Boston, MA, USA; ^6^ Department of Statistics, University of Virginia, Charlottesville, VA, USA; ^7^ Department of Epidemiology and Biostatistics, School of Public Health, Anhui Medical University, Hefei, China; ^8^ Division of Epidemiology and Biostatistics, University of Illinois at Chicago School of Public Health, Chicago, IL, USA

**Keywords:** LEP G2548A, LEPR Q223R, creatine kinase, hyperlipidemia, simvastatin

## Abstract

We investigated the associations of *LEP G2548A* and *LEPR Q223R* polymorphisms with statin-induced creatine kinase (CK) elevation among Chinese patients with hyperlipidemia. A total of587 enrolled individuals were treated with 20 mg/d oral simvastatin for 8 consecutive weeks. Genotyping of *LEP G2548A* and *LEPR Q223R* were conducted using PCR-RFLP. Multiple regression analyses showed that, in the Dongzhi region only, patients carrying the *LEP AA* genotype had a significantly greater increase in CK levels compared to those carrying the *AG+GG* genotypes after four weeks (*P* = 0.004) and eight weeks (*P* < 0.001) consecutive simvastatin treatment. Patients were further divided into three groups based on the tertiles of the CK distribution. Compared to subjects in the lowest tertile of CK elevation, the adjusted relative odds of having the AG+GG genotypes among subjects in the highest tertile was 0.5 (95% CI, 0.3 to 0.7) and 0.4 (95% CI, 0.2 to 0.6) after the fourth and eighth weeks, respectively. The interaction terms between the Beijing or Dongzhi region and the *LEP GA+AA* genotypes were marginally significant for CK elevation at the fourth week (*P* = 0.057) and significant for CK elevation at the eighth week (*P* = 0.002). The adverse effect of the *LEP G2548A* polymorphism on increasing CK levels may be dependent on the environmental milieu. It suggests that lifestyle interventions might offset the side effects of simvastatin therapy among those with genetic susceptibility. Further research is needed to identify specific individual-level factors for clinical practice that modify the effect of genotype.

## INTRODUCTION

3-hydroxy-3-methylglutaryl-coenzyme A (HMG-CoA) reductase inhibitors, commonly known as statins, are believed to inhibit cholesterol biosynthesis and are thus the most widely used cholesterol-lowering drugs to treat hypercholesterolemia [1, 2]. Statins disturb cholesterol biosynthesis by hindering the activity of HMG-CoA reductase in the rate-limiting step of cholesterol synthesis. This inhibition leads to increased levels of low-density lipoprotein (LDL) receptors, which results in increased uptake and degradation of low-density lipoprotein cholesterol (LDL-C), reduced cholesterol accumulation, and decreased lipoprotein secretion and cholesterol synthesis.

Despite efficiently lowering cholesterol levels, reducing clinical cardiovascular events by 20% to 50% [3], and having a positive benefit-risk ratio, statins are used with caution [4, 5] because they are known to induce adverse muscular events ranging from nonspecific myalgia to rhabdomyolysis. The Prediction of Muscular Risk in Observational Conditions study found that 10.5% of patients using statins experienced muscular symptoms [6]. Serum creatine kinase (CK) was often measured as a proxy to identify the severity of statin-induced myotoxicity. Moreover, for individuals with serum CK concentrations exceeding threefold the upper normal limit (UNL), CK levels and muscle damage have been closely related [7, 8]. Rhabdomyolysis, the most serious muscular event tied to statins, is caused by muscle fiber breakdown with dramatic elevations in CK exceeding 10×UNL. Though statins currently on the market do not lead to rhabdomyolysis as often, the disease prevalence is not insignificant. Atorvastatin is present in 12% of all cases, while simvastatin accounts for about 25% [9]. To date, studies are yet to identify biomarkers of increased CK elevation risk. Genetic variants are likely to provide some evidence for explaining the inter-individual variability of myotoxicity in response to simvastatin.

CK is an enzyme found in multiple tissues and cell types. CK catalyzes the conversion of creatine (Cr) and creates phosphocreatine (PCr) and adenosine diphosphate (ADP) using adenosine triphosphate (ATP). This is an important mechanism in the cellular energy supply chain for safeguarding in tissues with inconstant energy demands, namely skeletal and cardiac muscle [10]. CK and the adenosine 5′-monophosphate-activated protein kinase (AMPK) systems coordinate to maintain energy homeostasis [11]. The CK system provides a rapid response to energy challenges, while AMPK mediates medium and long-term adaptations [12]. Increased intracellular AMP : ATP and Cr : PCr ratios promote AMPK activation, which indicates insufficient cellular energy [13, 14]. AMPK then phosphorylates and inactivates the muscle-specific isoform of CK (MM-CK) [13]. The PCr system quickly replenishes ATP levels during energy-demanding processes. Evidence has shown that leptin can phosphorylate and activate AMPK [15]. Thus we speculate that leptin may play a role in regulating CK levels.

The *LEP* gene, located at chromosome 7q31.3 and consisting of three exons separated by two introns, encodes a 16 kDa protein, leptin. Leptin binds and activates its receptor, *LEPR*, in the hypothalamus. *LEPR* is a single transmembrane protein distributed in many types of tissues with the gene located at chromosome 1p31 in the hypothalamus [16]. The common *G2548A* polymorphism of the *LEP* gene has been tied to serum leptin and BMI in obese individuals [17, 18]. This polymorphism may alter leptin expression at the transcriptional level and induce the adipose secretion levels of the hormone [19]. Additionally, the *Q223R* polymorphism of the *LEPR* gene has been associated with leptin levels, BMI, fat mass and blood pressure [20].

Regardless of a genetic predisposition to complex traits such as vascular diseases and their therapeutic efficacy, much evidence has also shown that a healthy lifestyle, which includes not smoking, avoiding obesity, physical activity, and a healthy diet pattern, has markedly reduced rates of incident cardiovascular events [21, 22]. Moreover, genetic risk might be attenuated by a favorable lifestyle. Khera *et al*. analyzed data for participants in three prospective cohorts and one cross-sectional study to find that both genetic and lifestyle factors contribute to individual-level risk of coronary artery disease, but increased genetic risk can be offset largely by a healthy lifestyle [23].

In the present study, we became the first group to investigate the effects of the *LEP G2548A* and *LEPR Q223R* polymorphisms on the safety of statin-induced CK elevation in Chinese patients with primary hyperlipidemia, as well as their interactions with regions differing in economic and lifestyle factors.

## RESULTS

### Baseline clinical and epidemiologic characteristics

In total, we recruited 734 patients with primary hyperlipidemia, but excluded 147 subjects from Beijing without genotypic data due to the degradation of one batch of samples in storage. This left 587 patients with primary hyperlipidemia who were selected in this study. We compared the difference between the included and excluded Beijing subjects on the basic characteristics. The measured characteristics of participants from Beijing with genotype data versus those without genotype data are mostly similar, as shown in Supplementary Table 1. For the 587 observed patients, baseline clinical and epidemiologic characteristics grouped by region were shown in Table 1. For the variables age, BMI, WC, SBP, fasting glucose, HDL, LDL, and CK, significant differences were observed between the Beijing and Dongzhi cohorts. The distributions of gender, education, occupation, cigarette smoking, alcohol drinking and work intensity were also significantly different between Beijing and Dongzhi (all *P* < 0.001). However, no significant differences in DBP, TC and TG levels were detected across the two regions.

**Table 1:** Epidemiologic characteristics in beijing and dongzhi regions

Variables	Beijing(*N* = 195)	Dongzhi(*N* = 392)	*P* value
	Mean ± SD	Mean ± SD	
**Age (years)**	54.8 ± 9.4	51.2 ± 7.0	**<0.001**
**BMIa (kg/m^2^)**	24.9 ± 3.3	23.9 ± 2.8	**<0.001**
**WC (m)**	0.9 ± 0.1	0.9 ± 0.1	**0.043**
**SBP (mmHg)**	122.7 ± 15.9	129.1 ± 21.6	**<0.001**
**DBP (mmHg)**	78.7 ± 8.7	77.4 ± 11.6	0.175
**Fasting glucose (mmol/L)**	5.4 ± 0.7	5.8 ± 0.5	**<0.001**
**TG (mmol/L)**	2.0 ± 0.9	1.8 ± 0.9	0.106
**TC (mmol/L)**	6.5 ± 0.6	6.5 ± 0.6	0.699
**HDL-C (mmol/L)**	1.4 ± 0.3	2.0 ± 0.5	**<0.001**
**LDL-C (mmol/L)**	4.2 ± 0.6	3.7 ± 0.7	**<0.001**
**Baseline CK (μmol/L)**	88.9 ± 37.2	113.6 ± 49.0	**<0.001**
**CK elevation (4 weeks)**	7.5 ± 40.5	35.9 ± 76.8	**<0.001**
**CK elevation (8 weeks)**	9.5 ± 40.6	85.9 ± 115.0	**<0.001**
	***N*** **(%)**	***N*** **(%)**	
**Gender**			
Male	28 (14.4)	161 (41.1)	**<0.001**
Female	167 (85.6)	231 (58.9)	
**Education**			
High school or lower	103 (52.8)	374 (95.4)	**<0.001**
College or higher	92 (47.2)	18 (4.6)	
**Occupation**			
Farmer	1 (0.5)	256 (65.3)	**<0.001**
Non-farmer	194 (99.5)	136 (34.7)	
**Cigarette Smoking**			
No	169 (86.7)	277 (70.7)	**<0.001**
Yes	26 (13.3)	115 (29.3)	
**Alcohol drinking**			
No	170 (87.2)	289 (73.7)	**<0.001**
Yes	25 (12.8)	103 (26.3)	
**Work intensity**			
Light	165 (84.6)	127 (32.4)	**<0.001**
Moderate	25 (12.8)	179 (45.7)	
Heavy	5 (2.6)	86 (21.9)	

^*^t-tests and Pearson's χ^2^ tests were applied to continuous and categorical variables, respectively.

Abbreviations: SBP, systolic blood pressure; DBP, diastolic blood pressure; BMI, body mass index; WC, waist circumference; TG, triglycerides; TC, total cholesterol; HDL-C, high-density lipoprotein cholesterol; LDL-C, low-density lipoprotein cholesterol; CK, Creatine Kinase.

^a^BMI = weight/height^2^.

Bold values denoted significant results.

### Association between *LEP G2548A* and *LEPR Q223R* polymorphisms and baseline CK levels

The results were shown in Table 2. Multiple linear regression analysis demonstrated that neither the *LEP G2548A* nor the *LEPR Q223R* polymorphism had an effect on baseline CK in the Dongzhi or Beijing regions individually. Consistently, there was no effect modification by region in the associations of the *LEP G2548A* and *LEPR Q223R* polymorphisms with baseline CK levels, as shown in Supplementary Table 2.

**Table 2:** Association between LEP G2548A and LEPR Q223R polymorphisms and baseline CK levels stratified by region

Variable	Region	LEP	LEPR	*N*	Mean ± SD	Unadjusted			Adjusted		
						beta	se	*P* value	beta	se	*P* value
**Baseline CK**	**Dongzh**i	AA		202	116.09 ± 51.62	Ref.			Ref.	.	.
		GA		161	109.70 ± 43.67	−6.40	5.16	0.215	−5.08	4.88	0.298
		GG		29	117.31 ± 58.37	1.22	9.70	0.900	−4.33	9.38	0.644
		GA+GG		190	110.86 ± 46.12	−5.24	4.94	0.289	−4.97	4.67	0.287
			RR	317	113.25 ± 48.58	Ref.			Ref.	.	.
			RQ	69	115.23 ± 52.12	1.99	6.51	0.760	−9.87	5.77	0.087
			QQ	6	110.67 ± 43.91	−2.58	20.18	0.898	17.28	36.95	0.640
			RQ+QQ	75	114.87 ± 51.27	1.62	6.29	0.796	−3.62	6.00	0.546
**Baseline CK**	**Beijing**	AA		109	91.80 ± 37.97	Ref.			Ref.	.	.
		GA		79	85.98 ± 36.61	−5.82	5.46	0.286	−6.13	5.42	0.258
		GG		7	76.00 ± 30.84	−15.80	14.40	0.272	−12.71	14.21	0.370
		GA+GG		86	85.17 ± 36.12	−6.63	5.33	0.213	−6.69	5.28	0.205
			RR	136	91.72 ± 36.68	Ref.			Ref.	.	.
			RQ	58	81.86 ± 38.10	−4.31	6.23	0.488	−9.14	5.69	0.108
			QQ	1	109.00 ± 0.0	3.87	19.22	0.840	33.65	36.98	0.362
			RQ+QQ	59	82.32 ± 37.93	−9.41	5.75	0.101	−8.50	5.69	0.134

Adjusted for: age, sex, BMI, cigarette smoking, and alcohol drinking.

Bold values denoted statistically significant results.

### Association of *LEP G2548A* and *LEPR Q223R* polymorphisms with elevation of CK levels in response to simvastatin treatment

To further detect the effects of the *LEP G2548A* and *LEPR Q223R* polymorphisms on the elevation of CK levels in response to simvastatin treatment, we investigated the relationship between those genotypes and changes in CK levels after four and eight weeks of simvastatin treatment among patients with primary hyperlipidemia. The analysis results were shown in Table 3. In the Dongzhi region, both genotypes were associated with CK elevation after four weeks of treatment but only the *LEP G2548A* polymorphism was associated with it after eight weeks of treatment. Neither genotype was associated with CK elevation in the Beijing region at either time point. Consistently, testing the region variable as an interaction term in the models showed effect modification by region of the associations between genotype and CK elevation for *LEP G2548A* at both time points and for *LEPR Q223R* at four weeks (Supplementary Table 3).

**Table 3:** Association between LEP G2548A and LEPR Q223R polymorphisms and the elevation of CK levels after 4 weeks and 8 weeks’ simvastatin treatment stratified by region

Variable	Region	LEP	LEPR	*N*	Mean ± SD	Unadjusted			Adjusted		
						beta	se	*P* value	beta	se	*P* value
**At 4 wee**ks											
**CK elevatio**n	Dongzhi	AA		202	46.96 ± 93.69	Ref.	.	.	Ref.	.	.
		GA		161	22.14 ± 47.89	–24.81	8.01	**0.002**	–24.15	7.90	**0.002**
		GG		29	34.72 ± 66.02	–12.23	15.06	0.416	–9.70	15.17	0.522
		GA+GG		190	24.06 ± 51.07	–22.89	7.67	**0.003**	–22.00	7.57	**0.004**
			RR	317	28.53 ± 57.12	Ref.	.	.	Ref.	.	.
			RQ	69	68.61 ± 131.51	40.08	9.99	**<0.001**	36.08	10.02	**<0.001**
			QQ	6	46.50 ± 43.92	17.97	30.99	0.562	22.45	30.91	0.467
			RQ+QQ	75	66.84 ± 126.72	38.31	9.66	**<0.001**	34.93	9.66	**<0.001**
	Beijing	AA		109	7.21 ± 40.39	Ref.	.	.	Ref.	.	.
		GA		79	7.36 ± 41.23	0.15	5.96	0.980	0.47	6.00	0.937
		GG		7	12.29 ± 37.99	5.08	15.73	0.746	4.54	15.73	0.773
		GA+GG		86	7.76 ± 40.79	0.55	5.82	0.924	0.82	5.85	0.888
			RR	136	9.09 ± 42.02	Ref.	.	.	Ref.	.	.
			RQ	58	3.33 ± 36.91	–5.76	6.31	0.361	–6.12	6.32	0.332
			QQ	1	23.00 ± 0.0	13.91	40.40	0.730	12.41	41.03	0.762
			RQ+QQ	59	3.66 ± 36.68	–5.43	6.28	0.387	–5.84	6.29	0.353
**At 8 weeks**											
**CK elevation**	Dongzhi	AA		202	109.66 ± 136.89	Ref.	.	.	Ref.	.	.
		GA		161	59.20 ± 78.31	–50.46	11.85	**<0.001**	–50.39	11.83	**<0.001**
		GG		29	67.86 ± 81.05	–41.80	22.27	0.060	–39.29	22.72	0.083
		GA+GG		190	60.53 ± 78.58	–49.13	11.34	**<0.001**	–48.73	11.32	**<0.001**
			RR	317	84.76 ± 107.91	Ref.	.	.	Ref.	.	.
			RQ	69	89.35 ± 144.80	4.59	15.25	0.763	2.34	15.43	0.879
			QQ	6	102.83 ± 111.03	18.07	47.31	0.702	25.93	47.60	0.585
			RQ+QQ	75	90.43 ± 141.82	5.67	14.74	0.700	4.33	14.87	0.771
	Beijing	AA		109	9.20 ± 46.10	Ref.	.	.	Ref.	.	.
		GA		79	10.97 ± 33.49	1.77	5.97	0.766	2.61	6.01	0.664
		GG		7	-2.43 ± 16.00	–11.63	15.76	0.460	–11.49	15.74	0.465
		GA+GG		86	9.88 ± 32.57	0.68	5.84	0.907	1.41	5.86	0.809
			RR	136	9.19 ± 41.11	Ref.	.	.	Ref.	.	.
			RQ	58	9.97 ± 40.01	0.78	6.35	0.901	0.78	6.35	0.901
			QQ	1	25.00 ± 0.0	15.81	40.62	0.697	23.82	41.22	0.563
			RQ+QQ	59	10.23 ± 39.71	1.04	6.31	0.869	1.13	6.32	0.858

Adjusted for: age, sex, BMI, cigarette smoking, alcohol drinking, and baseline CK levels.

Bold values denoted statistically significant results.

### Association of *LEP G2548A* and *LEPR Q223R* polymorphisms with tertiles of CK elevation in response to simvastatin treatment

We classified all of the patients at each time point into three mutually exclusive clusters based on the tertiles of CK change within each region to evaluate associations between polymorphisms and CK change after treatment. We used binary genotypes as outcomes (justified by the results of Table 3) and predicted them by tertiles of CK elevation using logistic regression. As shown in Tables 4 and 5, the results in these models were consistent with those in Table 3. Specifically, in the Dongzhi region, tertiles of CK elevation were significantly associated with the *LEP G2548A* polymorphism at both four and eight weeks and with the *LEPR Q223R* polymorphism at four weeks only. The directions of these associations were consistent with those found in Table 3. In the Beijing region, there were no statistically significant associations between the tertiles of CK elevation and binary genotypes at either time point. Our models testing effect modification by region in these associations (Supplementary Tables 4 and 5) might have been underpowered due to the need to include two interaction terms for the three levels of CK elevation. There were no statistically significant interaction terms in these models except marginal significance for an effect modification by region (*p* = 0.06) for the first versus third tertiles of CK elevation and the *LEPR Q223R* polymorphism at four weeks.

**Table 4:** Associations of LEP G2548A genotype by tertiles of CK increase after simvastatin for 4 and 8 weeks stratified by region

Region	Tertile	CK increase Mean ± SD	Genotype GA+GG	AA	OR	Unadjusted			Adjusted	
						95%_CI	*P* value	OR	95%_CI	*P* value
**Dongzhi**	**At 4 weeks**									
	1st	–17.2 ± 25.3	78	54	1.0	.		1.0	.	
	2nd	21.7 ± 9.9	59	69	0.6	0.4–1.0	**0.036**	0.6	0.3–0.9	**0.027**
	3rd	102.6 ± 96.7	53	79	0.5	0.3–0.8	**0.002**	0.5	0.3–0.7	**0.002**
	**At 8 weeks**									
	1st	2.3 ± 22.4	80	49	1.0	.		1.0	.	
	2nd	54.9 ± 15.9	61	71	0.5	0.3–0.9	**0.011**	0.5	0.3–0.9	**0.013**
	3rd	199.2 ± 134.7	49	82	0.4	0.2–0.6	**<0.001**	0.4	0.2–0.6	**<0.001**
**Beijing**	**At 4 weeks**									
	1st	–23.4 ± 17.5	29	38	1.0	.		1.0	.	
	2nd	2.2 ± 4.3	30	34	1.2	0.6–2.3	0.680	1.0	0.4–2.1	0.901
	3rd	45.0 ± 47.1	27	37	1.0	0.5–1.9	0.899	0.8	0.4–1.7	0.534
	**At 8 weeks**									
	1st	–24.1 ± 23.5	26	38	1.0	.		1.0	.	
	2nd	4.8 ± 5.6	30	37	1.2	0.6–2.4	0.631	1.2	0.6–2.8	0.607
	3rd	48.0 ± 42.4	30	34	1.3	0.6–2.6	0.476	1.1	0.5–2.4	0.853

Adjusted for: age, sex, BMI, cigarette smoking, alcohol drinking, and baseline CK levels.

Bold values denoted statistically significant results.

**Table 5:** Associations of LEPRQ223R genotype by tertiles of CK increase after simvastatin for 4 and 8 weeks stratified by region

Region	Tertile	CK increase Mean ± SD	Genotype RQ+QQ	RR	OR	Unadjusted			Adjusted	
						95%_CI	*P* value	OR	95%_CI	*P* value
**Dongzhi**	**At 4 weeks**									
	1st	–17.2 ± 25.3	17	115	1.0			1.0		
	2nd	21.7 ± 9.9	24	104	1.56	0.79–3.07	0.196	1.42	0.68–2.95	0.349
	3rd	102.6 ± 96.7	34	98	2.35	1.24–4.46	**0.009**	2.19	1.13–4.26	**0.020**
	**At 8 weeks**									
	1st	2.3 ± 22.4	25	104	1.0	.		1.0	.	
	2nd	54.9 ± 15.9	22	110	0.83	0.44–1.57	0.568	0.72	0.37–1.39	0.321
	3rd	199.2 ± 134.7	28	103	1.13	0.62–2.07	0.689	1.16	0.62–2.15	0.646
**Beijing**	**At 4 weeks**									
	1st	–23.4 ± 17.5	23	44	1.0	.		1.0	.	
	2nd	2.2 ± 4.3	19	45	0.81	0.39–1.69	0.569	0.59	0.25–1.38	0.224
	3rd	45.0 ± 47.1	17	47	0.69	0.33–1.46	0.335	0.57	0.26–1.27	0.168
	**At 8 weeks**									
	1st	–24.1 ± 23.5	17	47	1.0	.		1.0	.	
	2nd	4.8 ± 5.6	18	49	1.02	0.47–2.20	0.968	0.98	0.40–2.40	0.967
	3rd	48.0 ± 42.4	24	40	1.66	0.78–3.51	0.186	1.62	0.71–3.68	0.251

Adjusted for: age, sex, BMI, cigarette smoking, alcohol drinking, and baseline CK levels.

Bold values denoted statistically significant results.

## DISCUSSION

Our present study is the first to indicate that the *LEP G2548A* and *LEPR Q223R* polymorphisms are associated with increasing CK levels after simvastatin treatment and that these associations can potentially be modified by environmental factors. Specifically, in the Dongzhi region, *LEP G2548A* was associated with CK increase after four and eight weeks of treatment, and *LEPR Q223R* was associated with CK increase after four weeks of treatment, though not after eight weeks. Neither genotype was associated with CK increase in the Beijing region. Furthermore, neither the *LEP G2548A* nor the *LEPR Q223R* genotype was associated with baseline CK levels in either region.

The heritability of CK levels has been estimated to be as high as 38% [24], while recent data places it at 19.33% [25]. A published genome-wide association study (GWAS) of 3,232,779 imputed variants in 3,412 statin users identified two missense variants that effected serum CK levels; one in CKM (rs11559024) and one in LILRB5 (rs12975366) [26]. Further work identified a variant in CD163 (rs7136716) to also be associated with serum CK levels [27]. To further search for sequence variants influencing serum CK levels, an Icelandic study of 28.3 million sequence variants identified through whole-genome sequencing of 2,636 Icelanders was imputed into 63,159 people with CK measurements [25]. This work found thirteen variants related to serum CK levels, of which eight variants were confirmed in the genes encoding the enzymes being measured (CKM, LILRB5 and CD163). However, our current data did not support the idea that the *LEP G2548A* and *LEPR Q223R* genotypes were associated with baseline CK levels.

Based on knowledge of the pharmacokinetic and pharmacodynamic processes involved in the effects of statins, it is evident that genetic polymorphisms may be useful in predicting adverse drug reactions to statin therapy. Several transporters involved in statin disposition are known to cause statin-induced myotoxicity. The main transporters are organic anion transporting polypeptide 1B1 coded by the SLCO1B1 gene, ATP-binding cassette (ABC) transporters subfamily B member 1 coded by the ABCB1 gene, and ABC transporters subfamily G member 2 coded by the ABCG2 gene. A recent pharmacogenetic study showed that statin-induced CK elevation is associated with polymorphisms in both the SLCO1B1 and ABCB1 genes [28]. In detail, the C allele in the T521C SNP of the SLCO1B1 gene and the T allele in the C1236T SNP of the ABCB1 gene exhibit elevated serum CK levels [29] and a higher risk of simvastatin- and rosuvastatin-induced myopathy [30–32]. In contrast, the G allele in the A388G SNP of the SLCO1B1 gene results in a lower risk of statin-induced myopathy [32–34].

Leptin is the most significant energy- and appetite-regulating peptide. It is produced predominantly in the adipose tissue and is proportionally related to adiposity. Thus, serum leptin concentrations can be used to determine the amount of energy reserves stored in adipose tissue [35]. Studies indicated that plasma leptin levels are higher in obese patients [36], and these levels dropped during fasting [37]. Additionally, leptin is a key regulator of feeding behavior and has been tied to hypertension, abdominal obesity, dyslipidemia and other metabolic risk factors in certain populations [38–41]. Numerous studies have reported that the *LEP G2548A* [42] and *LEPR Q223R* [16] polymorphisms were associated with lipid profiles, in which leptin may contribute to the cholesterol metabolic process [43]. Leptin down-regulates the hepatic activity of HMG-CoA reductase and up-regulates activity of both sterol 27-hydroxylase and cholesterol 7α-hydroxylase, which leads to decreased plasma very-low density lipoprotein cholesterol (VLDL-C) concentrations [44]. Moreover, leptin is also known to phosphorylate and activate AMPK via central and peripheral mechanisms. AMPK in peripheral tissues, including liver, muscle and adipose tissue, directly increases energy expenditure, and AMPK in the central nervous system indirectly alters peripheral energy expenditure [45]. AMPK is a protein kinase which phosphorylates and inactivates HMG-CoA reductase, so it helps inhibit the synthesis of cholesterol and triglycerides [46]. Therefore, leptin can competitively act with statins on inhibiting the HMG-CoA reductase, reducing the cholesterol synthesis and increasing serum CK levels.

CK levels vary largely in myopathic individuals and reflect muscle injury [47]. Muscular injury is characterized by impaired striated muscle cell integrity and the release of intracellular enzymes into the circulation in patients with statin therapy [48], especially CK and myoglobin [49]. Meyer zuSchwabedissen *et al*. [50] reported that patients who received fluvastatin and telmisartan treatment subsequently suffered myalgia along with elevated CK levels. Evidence has shown that both CK and AMPK systems coordinate in maintaining energy homeostasis [11], and leptin can phosphorylate and activate AMPK [15]. Thus, we speculate that leptin may contribute to regulating CK levels. Our results showed that, after four consecutive weeks of simvastatin treatment, patients in the Dongzhi region carrying the *AA* genotype of *LEP G2548A* had a significantly higher increase in CK levels than those with the *GA* (*P* = 0.002) or *GA+GG* genotypes (*P* = 0.004). Patients carrying the *RQ+QQ* genotype of the *LEPR Q223R* gene had a significantly higher increase in CK levels than those with *RR* (*P* < 0.001). After eight weeks of simvastatin treatment, patients carrying the *AA* genotype of *LEP G2548A* had a significantly higher increase in CK levels than those with the *GA* (*P* < 0.001) or *GA+GG* genotypes (*P* < 0.001). However, there was not a significant relationship between *LEPR Q223R* and CK elevation after eight weeks of treatment in the Dongzhi patients. Furthermore, no significant differences in the elevation of CK levels were observed among different *LEPR Q223R* genotypes after both four and eight weeks of simvastatin treatment in Beijing regional patients.

CK elevation in response to simvastatin is a complex multifactorial trait. Generally, it is attributed to an interaction between a patient's genetic background and various environmental factors. In the present study, our populations were sampled randomly within the two regions Dongzhi and Beijing. Dongzhi and Beijing are located in the south and north of China, respectively, and residents of the two places have their own distinctive customs and cultures including geographical location, intra-area marriages, dietary habits and lifestyles. Contrary to the Dongzhi region, the Beijing region showed higher percentages of male, educated and working individuals, but lower prevalence of cigarette smoking, alcohol drinking and work intensity. The reason for these discrepancies between the two regions may be the dramatically distinct cultural and economic environments. Beijing is the national capital city and in northern China, but Dongzhi is a rural area in southern China. Our data showed that the interaction term of Beijing region × *LEP (GA+GG)* genotypes was marginally significant for CK elevation (*P* = 0.057) at the fourth week and strongly significant for CK elevation (*P* = 0.002) at the eighth week. Supplementary Table 3 also supported that the *LEP* genotype was associated with CK increase in Dongzhi after four and eight weeks of treatment, though not in Beijing. These differences between Dongzhi and Beijing, shown in the effect modification by region, were statistically significant. The *LEPR* genotype was associated with CK increase in Dongzhi after 4 weeks of treatment, but not in Beijing. However, after 8 weeks of treatment, the *LEPR* genotype no longer had any association with CK increase in either region. The difference in the association between Dongzhi and Beijing at four weeks was statistically significant.

As mentioned above, there are many differences between Beijing and Dongzhi. An interesting possibility raised by this paper is that, in addition to genes, gene-environment interactions can also affect the side effects of simvastatin. This opens the door to the possibility that side effects could be limited not only by individualized medicine based on genotype, but also by lifestyle changes recommended according to genotype. In other words, correcting certain lifestyle factors could reduce the adverse effects of genotype on the likelihood of side effects from pharmaceuticals. Similar to our finding, unfavorable lifestyle factors and genetic variants were determined to be of the same importance in contributing to increased risk of coronary artery disease [23]. Among participants at high genetic risk, a favorable lifestyle was associated with a nearly 50% lower relative risk of coronary artery disease than was an unfavorable lifestyle. The lifestyle factors that are interacting with genotype to affect CK increase in this study are unknown currently because we used region as the proxy for lifestyle. Our region variable is associated with many different lifestyle factors. The next step in our analysis will be to look at all of the lifestyle-related variables that have different distributions in Beijing compared to Dongzhi and investigate which of these variables modify the associations of the genotypes with CK increase. The variables included in this study that have different prevalence or averages in Beijing versus Dongzhi were age, gender, BMI, WC, SBP, fasting glucose, HDL, LDL, CK, education, occupation, cigarette smoking, alcohol consumption and work intensity. We will investigate whether these are effect modifiers in the association between genotypes and CK increase, regardless of region, in a future analysis.

We excluded 147 people from Beijing because their genotype data was unreliable due to degradation of one batch of samples in storage. The measured characteristics of participants from Beijing with genotype data versus those without genotype data are mostly similar. Among the seventeen variables included in Supplementary Table 1, only three show significant differences between these two groups. Furthermore, for these three variables, the magnitudes of differences between groups are not especially large, although still statistically significant. Beyond this, even though these three variables significantly differ between the two groups, this does not indicate that they are significant effect modifiers of the associations between genotypes and CK increase, a condition that would be necessary for our Beijing results to suffer selection bias due to this loss of genotype data. Nonetheless, the results we found in our Beijing participants should be read with this loss of data in mind and awareness that we cannot completely discount selection bias due to this loss.

Two limitations of our study should be mentioned. As is well known, leptin plays an important role in the relationship of *LEP G2548A* and *LEPR Q223R* with obesity, diabetes mellitus, dyslipidemia and CK. We did not measure leptin levels, so we could not determine whether the *LEP* and *LEPR* polymorphisms were associated with leptin levels or whether the *LEP* and *LEPR* polymorphisms and leptin levels interacted in their associations with the outcomes in this study. Limited evidence showed that neither *LEP G2548A* nor *LEPR Q223R* polymorphisms were significantly associated with plasma leptin levels in Chinese populations [51, 52]. Therefore, we are considering to measure serum leptin, adipokine and soluble leptin receptor concentrations in our ongoing large scale cardiovascular cohort project. Additionally, all patients received simvastatin treatment orally at the low dosage of 20 mg/d for eight consecutive weeks. We cannot dichotomize the CK concentration as CK elevation or no CK elevation based on the common criteria of 3×UNL CK or higher, as only a few subjects met this criterion; three and seventeen participants met it for the response to CK elevation after four and eight weeks of treatment, respectively. Also, we had to exclude some patients in the Beijing area due to unreliable genotype data. Although the characteristics of included and excluded patients from Beijing were similar and so the likelihood of selection bias was reduced, a limitation was that we had less power to detect statistically significant associations in the Beijing region compared to Dongzhi.

In conclusion, our major findings demonstrated that the *LEP G2548A* (after four and eight weeks of treatment) and *LEPR Q223R* polymorphisms (after four weeks treatment) were associated with simvastatin-induced CK increase in the Dongzhi region, though not in Beijing. This opens the possibility that lifestyle interventions could reduce or eliminate some of the adverse side effects of statin therapy that are associated with particular genotypes.

## MATERIALS AND METHODS

### Study population

In total, 734 Chinese subjects with primary hyperlipidemia were enrolled from Beijing and Dongzhi, Anhui Province. 587 patients with complete information on genotypes and other parameters were included in this study. We screened eligible patients with hyperlipidemia through medical history, physical examination and clinical laboratory evaluation, including the lipid profile. No participant demonstrated symptomatic ischemic heart disease or any other vascular diseases. Patients were required to stop taking any lipid-, hypertension- or glucose-lowering drugs at least seven days prior to the study. All participants gave informed consent, and the study protocol was approved by the ethics committee of the Institute of Biomedicine at Anhui Medical University.

Participants who met the following criteria of fasting serum lipid levels were deemed as having primary hyperlipidemia: TC 5.72–8.32 mmol/L or LDL-C 3.64–6.50 mmol/L and TG at least 1.70 mmol/L [53, 54]. To avoid any potential side effects from our treatment program, patients with any of the following characteristics were excluded: (1) impaired hepatic function (aminotransferase levels greater than two times the norm coupled with a history of chronic liver disease, such as cirrhosis or alcohol abuse), (2) impaired renal function (serum creatine levels greater than 1.8 mg/dL or a history of chronic renal disease, such as glomerulonephritis, chronic pyelonephritis, obstructive renal disease, or proteinuria), (3) raised thyroid-stimulating hormone (TSH) levels (greater than 5.0 μU/L), and (4) any other medical conditions that might preclude successful completion.

### Simvastatin treatment

Following a washout period of seven to ten days, all patients began oral treatment of a fixed 20 mg/d dosage of simvastatin for eight consecutive weeks. Patients administered the drugs themselves nightly between 8 p.m. and 10 p.m. During the study period, subjects visited our clinical center biweekly to obtain their drugs for the next two weeks and to report any adverse effects. Patients whose laboratory parameters were affected by the treatment were excluded during the study. At baseline, four weeks, and eight weeks, blood samples were drawn following a fourteen hour overnight fast in order to determine serum lipids levels and other biochemical parameters, such as TG, TC, HDL-C, LDL-C, fasting glucose and CK.

### Laboratory determinations

After a fourteen hour overnight fast, venous blood samples were taken the following morning between 8 a.m. and 10 a.m. and stored in ethylenediaminetetraacetic acid (EDTA) tubes. The samples were then centrifuged at 2500 r/min for ten min in order to obtain the serum. In our analytical center, automated biochemical analysis was used for the laboratory determinations. Levels of TG, TC, HDL-C, LDL-C and CK were calculated with enzymatic colorimetric assays (Roche Diagnostics, Mannheim, Germany). Fasting glucose levels were determined using the glucose oxidase method. The intra-assay and inter-assay coefficients of variation were less than 5% for all assays performed.

### Genotyping of LEP G2548A and LEPR Q223R

DNA was extracted from EDTA-treated whole blood and stored at –20°C. Genotyping of the *LEP G2548A* (rs7799039) and *LEPR Q223R* (rs1137101) genes was carried out using the polymerase chain reaction-restriction fragment length polymorphism (PCR-RFLP) assay. For the *LEP G2548A* gene, the primer-pairs for PCR amplification were forward 5′-TTTCCTGTAATTTTCCCGTGAG-3′ and reverse 5′-AAAGCAAAGACAGGCATAAAAA-3′. For the *LEPR Q223R* gene, they were forward 5′-ACCTCTGGTTCCCCAAAAAG-3′ and reverse 5′-TCATCATTTTAGTGCATAACTTACCC-3′. PCR amplification was carried out in 25 μL PCR mixture in a PCR Amplifier (Long Gene). For the *LEP G2548A* gene, the process was as follows: an initial denaturation at 95°C for 60 s, followed by 35 cycles of denaturation at 95°C for 30 s, annealing at 56°C for 45 s, and extension at 72°C for 30 s, with a final extension at 72°C for 7 min. For the *LEPR Q223R* gene, the process was as follows: an initial denaturation at 94°C for 2 min, followed by 35 cycles of denaturation at 94°C for 30 s, annealing at 56°C for 45 s, and extension at 72°C for 45 s, then a final extension at 72°C for 6 min. After PCR amplification, 15 μL was digested with restriction enzymes: HhaI [55] for *LEP* at 37°C for 4 h, and MspI [55] for *LEPR* at 37°C for 5 h. The digestion products were then separated on 3% agarose gels stained with ethidium bromide. The 242bp fragment was divided into 242bp, 181bp and 61bp; the 212bp fragment was divided into 212bp, 151bp and 61bp. All sample sets genotyped for each SNP in our present study had overall call rates of 95% after excluding samples that consistently failed. Then we selected around 10% samples for repeated genotyping. We had 100% concordance of repeated samples in these quality control tests..

### Statistical analysis

Continuous data were presented as mean ± standard deviation (SD), and categorical data were presented as proportions. Comparisons between groups were performed using chi-square tests for categorical variables and Student's *t* test for continuous variables. Multiple linear/logistic regression models were fit to estimate the associations between *LEP* and *LEPR* polymorphisms and CK levels at baseline and after simvastatin treatment. All analyses conducted were stratified by region. The regression coefficients, listed as beta values in our tables, were estimated using the least-squares method and can be interpreted as the mean difference in CK elevation between a genotype group and the reference group. We also examined interactions on the multiplicative scale between the single-nucleotide polymorphisms and the different environmental regions in association with CK elevation. The SAS 8.0 software package (SAS Institute, Cary, NC) and IBM SPSS software package (version 19.0 for windows; IBM Inc. Armonk, NY, USA) were used for statistical analysis. *P*-values less than 0.05 were considered to be significant.

## SUPPLEMENTARY MATERIALS TABLES


