# Supplementary material for: Effect modification by region in the associations of LEP G2548A and LEPR Q223R polymorphisms with statin-induced CK elevation
Source: Oncotarget. 2017 Nov 18;8(64):107565–76. doi: 10.18632/oncotarget.22506 (PMC5746089; doi:10.18632/oncotarget.22506)
Supplement: Supplementary file 1 [file oncotarget-08-107565-s001.pdf]

Effect modification by region in the associations of *LEP G2548A* and *LEPR Q223R* polymorphisms with statin-induced CK elevation

SUPPLEMENTARY MATERIALS

Supplementary Table 1: Associations of epidemiologic characteristics among Beijing participants with presence or absence of genotype data

Variables	Beijing included (*N* = 195)	Beijing excluded due to missing genotype data (*N* = 147)	*P* value
	Mean ± SD	Mean ± SD	
**Age (years)**	54.8 ± 9.4	56.0 ± 9.3	0.243
**BMIa (kg/m^2)**	24.9 ± 3.3	24.8 ± 3.0	0.935
**WC (m)**	0.9 ± 0.1	0.9 ± 0.1	0.292
**SBP (mmHg)**	122.7 ± 15.9	126.1 ± 16.3	0.052
**DBP (mmHg)**	78.7 ± 8.7	78.4 ± 10.2	0.776
**Fasting glucose (mmol/L)**	5.4 ± 0.7	5.6 ± 0.6	**0.017**
**TG (mmol/L)**	2.0 ± 0.9	1.9 ± 0.9	0.674
**TC (mmol/L)**	6.5 ± 0.6	6.5 ± 0.7	0.645
**HDL-C (mmol/L)**	1.4 ± 0.3	1.4 ± 0.3	0.543
**LDL-C (mmol/L)**	4.2 ± 0.6	4.0 ± 0.6	**0.016**
**Baseline CK (µmol/L)**	88.9 ± 37.2	90.7 ± 41.3	0.666
	N (%)	*N* (%)	
**Gender**			
Male	28 (14.4)	24 (16.2)	0.635
Female	167 (85.6)	123 (83.8)	
**Education**			
High school or lower	103 (52.8)	91 (61.5)	0.109
College or higher	92 (47.2)	56 (38.5)	
**Occupation**			
Farmer	1 (0.5)	7 (5.4)	**0.005**
Non-farmer	194 (99.5)	140 (94.6)	
**Cigarette Smoking**			
No	169 (86.7)	125 (85.1)	0.686
Yes	26 (13.3)	22 (14.9)	
**Alcohol drinking**			
No	170 (87.2)	132 (89.9)	0.443
Yes	25 (12.8)	15 (10.1)	
**Work intensity**			
Light	165 (84.6)	111 (75.7)	0.082
Moderate	25 (12.8)	27 (18.2)	
Heavy	5 (2.6)	9 (6.1)	

[*] *t*-tests and Pearson's χ^2 tests were applied to continuous and categorical variables, respectively.
Abbreviations: SBP, systolic blood pressure; DBP, diastolic blood pressure; BMI, body mass index; WC, waist circumference; TG, triglycerides; TC, total cholesterol; HDL-C, high-density lipoprotein cholesterol; LDL-C, low-density lipoprotein cholesterol; CK, Creatine Kinase.
[a] BMI = weight/height^2.
Bold values denoted significant results.

Supplementary Table 2: Effect modification by region in the association between LEP G2548A and LEPR Q223R polymorphisms and baseline CK levels

Variables	LEP	LEPR	Adjusted		
			**beta**	**se**	***P* value**
**Baseline CK**	LEP AA		0.0	.	.
	LEP GA+GG		–5.12	4.38	0.242
	Dongzhi		0.0	.	.
	Beijing		–17.37	5.39	**0.001**
	LEP (GA+GG)*Beijing		–2.04	7.65	0.790
**Baseline CK**		LEPR RR	0.0	.	.
		LEPR RQ+QQ	–3.13	5.62	0.576
		Dongzhi	0.0	.	.
		Beijing	–16.41	4.64	**<0.001**
		LEPR(RQ+QQ)*Beijing	–4.42	8.82	0.616

Adjusted for: age, sex, BMI, cigarette smoking, and alcohol drinking.
Bold values denoted statistically significant results.

Supplementary Table 3: Effect modification by region in the association between LEP G2548A and LEPR Q223R polymorphisms and the elevation of CK levels after 4 weeks and 8 weeks' simvastatin treatment

Variables	LEP	LEPR	Adjusted		
			**beta**	**se**	***P* value**
**At 4 weeks**	LEP AA		0.0	.	.
**CK elevation**	LEP GA+GG		–22.4	6.7	**0.001**
	Dongzhi		0.0	.	.
	Beijing		–34.8	8.2	**<0.001**
	LEP (GA+GG)*Beijing		22.1	11.6	**0.057**
		LEPR RR	0.0	.	.
		LEPR RQ+QQ	36.21	8.47	**<0.001**
		Dongzhi	0.0	.	.
		Beijing	–17.21	6.97	**0.013**
		LEPR(RQ+QQ)*Beijing	–41.19	13.30	**0.002**
**At 8 weeks**	LEP AA		0.0	.	.
**CK elevation**	LEP GA+GG		–47.7	9.5	**<0.001**
	Dongzhi		0.0	.	.
	Beijing		–98.2	11.8	**<0.001**
	LEP (GA+GG)*Beijing		51.4	16.6	**0.002**
		LEPR RR	0.0	.	.
		LEPR RQ+QQ	5.17	12.50	0.6793
		Dongzhi	0.0	.	.
		Beijing	–78.48	10.28	**<0.001**
		LEPR(RQ+QQ)*Beijing	–3.75	19.62	0.8486

Adjusted for: age, sex, BMI, cigarette smoking, alcohol drinking, and baseline CK levels.
Bold values denoted statistically significant results.

Supplementary Table 4: Effect modification by region in the associations of LEP G2548A genotype by tertiles of CK increase after simvastatin for 4 and 8 weeks stratified by region

Variables	Interaction	Adjusted		
		beta	se	*P* value
**At 4 weeks**	LEP AA	1.00	.	
**1st vs. 2nd**	LEP GA+GG	0.48	0.27–0.86	**0.013**
	Dongzhi	1.00	.	
	Beijing	0.27	0.14–0.52	**<0.001**
	LEP (GA+GG)*Beijing	1.90	0.79–4.55	0.149
**1st vs. 3rd**	LEP AA	1.00	.	
	LEP GA+GG	0.39	0.23–0.66	**<0.001**
	Dongzhi	1.00	.	
	Beijing	0.13	0.06–0.26	**<0.001**
	LEP (GA+GG)*Beijing	1.91	0.71–5.18	0.2015
**At 8 weeks**	LEP AA	1.00	.	
**1st vs. 2nd**	LEP GA+GG	0.66	0.36–1.20	0.1698
	Dongzhi	1.00	.	
	Beijing	0.18	0.09–0.35	**<0.001**
	LEP (GA+GG)*Beijing	1.79	0.75–4.28	0.1915
**1st vs. 3rd**	LEP AA	1.00	.	
	LEP GA+GG	0.37	0.21–0.64	**<0.001**
	Dongzhi	1.00	.	
	Beijing	0.03	0.01–0.08	**<0.001**
	LEP (GA+GG)*Beijing	1.00	0.24–4.26	0.9978

Adjusted for: age, sex, BMI, cigarette smoking, alcohol drinking, and baseline CK levels.
Bold values denoted statistically significant results.

Supplementary Table 5: Effect modification by region in the associations of LEPR Q223R genotype by tertiles of CK increase after simvastatin for 4 and 8 weeks stratified by region

Variables	Interaction	Adjusted		
		beta	se	*P* value
**At 4 weeks**	LEPR RR	1.00	.	
**1st vs. 2nd**	LEPR RQ+QQ	1.06	0.47–2.40	0.890
	Dongzhi	1.00	.	
	Beijing	0.42	0.25–0.71	**0.001**
	LEPR(RQ+QQ)*Beijing	0.77	0.26–2.28	0.639
**1st vs. 3rd**	LEPR RR	1.00	.	
	LEPR RQ+QQ	2.27	1.14–4.55	**0.020**
	Dongzhi	1.00	.	
	Beijing	0.24	0.13–0.43	**<0.001**
	LEPR(RQ+QQ)*Beijing	0.33	0.10–1.06	0.062
**At 8 weeks**	LEPR RR	1.00	.	
**1st vs. 2nd**	LEPR RQ+QQ	1.17	0.55–2.52	0.683
	Dongzhi	1.00	.	
	Beijing	0.23	0.13–0.39	**<0.001**
	LEPR(RQ+QQ)*Beijing	1.25	0.45–3.49	0.672
**1st vs. 3rd**	LEPR RR	1.00	.	
	LEPR RQ+QQ	1.15	0.56–2.37	0.709
	Dongzhi	1.00	.	
	Beijing	0.04	0.02–0.08	**<0.001**
	LEPR(RQ+QQ)*Beijing	1.35	0.34–5.39	0.669

Adjusted for: age, sex, BMI, cigarette smoking, alcohol drinking, and baseline CK levels.
Bold values denoted statistically significant results.
